# The effect of exercise training and physiotherapy on left and right heart function in heart failure with preserved ejection fraction: a systematic literature review

**DOI:** 10.1007/s10741-022-10259-1

**Published:** 2022-07-13

**Authors:** Eglė Palevičiūtė, Toma Šimbelytė, Christina A. Eichstaedt, Nicola Benjamin, Benjamin Egenlauf, Ekkehard Grünig, Jelena Čelutkienė

**Affiliations:** 1 Clinic of Cardiac and Vascular Diseases, Institute of Clinical Medicine, Faculty of Medicine, Vilnius University, Santariskiu-2, 08661 Vilnius, Lithuania; 2 Centre for Pulmonary Hypertension, Thoraxklinik Heidelberg gGmbH at Heidelberg University Hospital, Translational Lung Research Center Heidelberg (TLRC), German Center for Lung Research (DZL), Heidelberg, Germany; 3 Laboratory for Molecular Genetic Diagnostics, Institute of Human Genetics, Heidelberg University, Heidelberg, Germany

**Keywords:** Exercise training, Physiotherapy, Heart failure with preserved ejection fraction, Cardiac imaging, Diastolic function

## Abstract

The impact of exercise training and physiotherapy on heart function and pulmonary circulation parameters in heart failure with preserved ejection fraction (HFpEF) patients is uncertain. Hence, we performed a systematic review of published trials studying physical training in HFpEF population, with a focus on exercise and physiotherapy effect on left ventricular (LV), right ventricular (RV) morphological, functional, and pulmonary circulation parameters. We searched Cochrane Library and MEDLINE/PubMed for trials that evaluated the effect of exercise training and/or physiotherapy in adult HFpEF patients (defined as LVEF ≥ 45%), including publications until March 2021. Our systematic review identified eighteen articles (*n* = 418 trained subjects, 4 to 52 weeks of training) and covered heterogeneous trials with various populations, designs, methodologies, and interventions. Five of twelve trials revealed a significant reduction of mitral E/e’ ratio after the training (− 1.2 to − 4.9). Seven studies examined left atrial volume index; three of them showed its decrease (− 3.7 to − 8 ml/m^2^). Findings were inconsistent regarding improvement of cardiac output, E/A ratio, and E wave DecT and uncertain for RV function and pulmonary hypertension parameters. For now, no reliable evidence about rehabilitation effect on HFpEF cardiac mechanisms is available. There are some hypotheses generating findings on potential positive effects to parameters of LV filling pressure (E/e’), left atrium size, cardiac output, and RV function. This encourages a broader and more complex assessment of parameters reflecting cardiac function in future HFpEF exercise training studies.

**Supplementary Information:**

The online version contains supplementary material available at 10.1007/s10741-022-10259-1.

## Introduction

Heart failure with preserved ejection fraction (HFpEF) accounts for approximately half of all heart failure (HF) patients and its burden is increasing [1]. A definite diagnosis of HFpEF can be made by right heart catheterization with pulmonary arterial wedge pressure (PAWP) ≥ 15 mmHg or left ventricular end diastolic pressure (LVEDP) ≥ 16 mmHg at rest in presence of preserved left ventricular systolic function. The hallmark of HFpEF is an elevation in left-sided filling pressures. In some patients, this leads to secondary pulmonary hypertension (PH). Pulmonary arterial pressure (PAP) is a marker of the severity and chronicity of pulmonary venous congestion/hypertension in HFpEF, and, if present, PH is associated with more pronounced symptoms and a poorer outcome [2, 3]. Consistent scientific data show that properly designed exercise interventions alone or as a component of comprehensive cardiac rehabilitation program for HF improved patients’ exercise capacity, symptoms, and health-related quality of life (QOL) and reduced the risk of all-cause and HF hospitalizations [4–7]. Of note, patients with HF with reduced ejection fraction (HFrEF) were predominant in most of the studies. European Society of Cardiology (ESC) guidelines for the diagnosis and treatment of acute and chronic HF recommend exercise rehabilitation not only to improve exercise capacity and QOL, but also to reduce HF hospitalizations, regardless of LV systolic function (Class I recommendation) [4].

The positive impact of exercise training on the functional capacity of HF patients is complex and can be determined by different pulmonary, cardiovascular, skeletal muscle, and metabolic adaptations that increase oxygen delivery and energy production [8]. As shown in several meta-analyses of randomized controlled trials (RCT), aerobic exercise training, especially long-term duration (≥ 6 months), reverses left ventricular (LV) remodeling in clinically stable patients with HFrEF [9–11]. Exercise training was associated with a significant improvement in LV ejection fraction (LV EF), when data from all RCT were pooled in the first meta-analysis (articles were published in 1966–2006; 14 trials; 812 patients) [9]. Later, this finding was confirmed in two updated meta-analyses (including articles published in 2006–2011: 16 trials with 813 patients, and 2007–2017: 18 trials with 1077 patients) [10, 11]. Moreover, in two meta-analyses, aerobic training led to significant improvements in LV end diastolic volume (EDV) (5 trials with 371 patients and 12 trials with 573 patients) and LV end systolic volume (ESV) (5 trials with 371 patients and 11 trials with 548 patients) [10, 11]. There are limited data on the effects of HFrEF patients exercising on more precise structural and functional echocardiographic parameters, such as myocardial velocities, strain and strain rate, stroke volume, right ventricular 3D ejection fraction, estimated systolic pulmonary arterial pressure, and size and collapsibility of inferior vena cava. This knowledge gap is even wider in HFpEF patients.

Earlier HFpEF rehabilitation systematic reviews and meta-analyses demonstrated positive impact of exercise training on functional capacity change, by improving peak oxygen uptake [12–17] and six-minute walk test distance [14, 15]. Moreover, HFpEF training seemed to be safe [12–16, 18, 19] and beneficial for the QOL of patients [12–17]. Five previous meta-analyses assessed the influence of exercising on only several echocardiographic parameters, mostly the mitral E/A ratios, E/e′ ratios, and E wave deceleration time (DecT), and their results were inconsistent [12–16]. Neither of prior systematic reviews evaluated the changes of right ventricular (RV) and pulmonary circulation parameters after the training. The summary of their findings is presented in Table 1.

**Table 1:** Results from previous HFpEF exercise training meta-analyses: the changes of left ventricular function and morphology, comparing exercise training vs. control groups

**Meta-analysis**	**Designs of included studies, *n***	**Participants (*n*) (training/control)**	**Results of specific echocardiographic parameter meta-analysis with exercise versus control**
Taylor et al. [12]	1 –observational 1 – non-RCT 1 – RCT	102/38	E/e′: − 0.9, 95% *CI*: − 3.8 to 2.0, *P* = 0.53^*^; random effect
	1 –observational 1 – non-RCT 1 – RCT	84/45	E/A: − 0.02, 95% *CI*: − 0.11 to 0.06, *P* = 0.56^*^; fixed effect
	1 –observational 1 – RCT	52/24	LV EDV (ml): 4.5, 95% *CI*: − 1.8 to 10.9, *P* = 0.16^*^; fixed effect
	1 –observational 2 – RCT	96/44	LV EF (%): 0.02, 95% *CI*; − 1.6 to 1.7, *P* = 0.98^*^; fixed effect
Pandey et al. [13]	4 – RCT	82/81	E/A: 0.08, 95% *CI*: − 0.01 to 0.16, *P* = 0.08^#^; fixed effect
	3 – RCT	62/70	DecT (ms): 2.92, 95% *CI*: − 18.56 to 24.41, *P* = 0.79^#^; fixed effect
	5 – RCT	126/111	LV EF (%): 1.26, 95% *CI*: − 0.13 to 2.66%, *P* = 0.08^#^; fixed effect
Dieberg et al. [14]	4 – RCT	85/60	E/e′: − 2.3, 95% *CI*: − 3.44 to − 1.19, *P* < 0.0001^*^; fixed effect
	3 – RCT	56/52	E/A: 0.07, 95% *CI*: 0.02 to 0.12, *P* = 0.005^*^; fixed effect
	3 – RCT	56/52	DecT (ms): − 13.2, 95% *CI*: − 19.8 to − 6.5, *P* = 0.0001^*^; fixed effect
Chan et al. [15]	5 – RCT	115/89	E/e′: − 2.38, 95% *CI*: − 3.47 to − 1.28, *P* < 0.0001^*^, fixed effect
	4 – RCT	86/81	E/A: + 0.07, 95% *CI*: 0.02 to 0.12, *P* = 0.006^*^, fixed effect
	3 – RCT	56/52	DecT (ms): − 13.2, 95% *CI*: − 19.8 to − 6.5, *P* = 0.0001^*^, fixed effect
Fukuta et al. [16]	4 – RCT	132/109	E/e′: − 1.20, 95% *CI*: − 4.07 to 1.66, *P* = 0.41^#^, random effect
	5 – RCT	128/124	E/A: 0.03, 95% *CI*: − 0.02 to 0.08, *P* = 0.27^#^; random effect
	3 – RCT	102/79	e′ (cm/s): 0.49, 95% *CI*: − 1.28 to 2.25, *P* = 0.59^#^; random effect
	3 – RCT	62/69	DecT (ms): − 2.04, 95% *CI*: − 26.53 to 22.45, *P* = 0.87^#^; random effect
	4 – RCT	140/120	LV EDV: − 0.03, 95% *CI*: − 0.28 to 0.21, *P* = 0.78^#^; fixed effect
	3 – RCT	116/90	LV mass: 0.07, 95% *CI*: − 0.21 to 0.35, *P* = 0.61^#^; fixed effect
	7 – RCT	202/174	LV EF: 0.85, 95% *CI*: − 0.128 to 1.83, *P* = 0.09^#^; fixed effect

^*^Mean difference; ^#^weighted mean difference

A single center exercise invasive hemodynamic study revealed that patients with HFpEF, complicated with PH and pulmonary vascular disease, demonstrate unique hemodynamic limitations during exercise that constrain aerobic capacity, including impaired recruitment of LV preload due to excessive right heart congestion (due to afterload) and blunted RV systolic reserve [20]. These conditions are leading to RV and pulmonary artery (PA) uncoupling with further limitation of exercise capacity and poor outcome [21].

In healthy subjects, intensive exercise has already shown to cause potentially deleterious remodeling of the RV [22, 23]. As pointed out by Arena et al. there may thus be an exercise training volume/intensity which may be detrimental to the RV in patients with HF and concomitant PH [24].

It is not clear whether changes of heart function and pulmonary circulation parallel improvement in cardiorespiratory fitness, or maybe exercise training may lead to harmful effects or worsening of the disease. We aimed to systematically review existing data on the impact of exercise and physiotherapy in HFpEF trials on LV, RV morphological, functional, and pulmonary circulation parameters.

## Methods

We prepared this article by following the PRISMA (Preferred Reporting Items for Systematic reviews and Meta-Analyses) guidelines [25].

We conducted a Cochrane Library and MEDLINE/PubMed search for all types of trials that evaluated the effects of various types of exercise training and/or physiotherapy in adult (> 18 years) HFpEF patients (defined as LVEF ≥ 45%), including all papers published from December 1991 to March 2021. Studies that merely enrolled patients with other cardiac or respiratory diseases were excluded. HFpEF data was extracted from studies if various parameters were included and reported separately. The main outcomes of interest were any reported echocardiographic, MRI, and invasive hemodynamic parameters.

For each database search, we used two groups of keywords and their synonyms for participants and intervention. The search strategy for MEDLINE/PubMed can be found in the Supplementary Material (Table S1). We modified search strategies according to each database to achieve the broadest research.

The search was limited to human studies only, adults (> 18 years), and the results were filtered by “Clinical Trial,” “Meta-Analysis,” and “Systematic Review.” Additionally, for any potential eligible trials, we manually searched in clinicaltrials.gov, Google Scholar, and the references of the identified studies.

Each title and abstract were independently evaluated by 2 reviewers. If at least one of the reviewers considered the trial to be eligible, it was obtained for primary analysis. After initial review, the full texts of selected studies were assessed to verify eligibility criteria.

Two reviewers assessed methodological quality of studies using modified Downs and Black Quality checklist, which is meant to assess the quality for both randomized and non-randomized trials [26]. The sub-domain, estimating the power, was modified (if the study conducted a power analysis to determine the sample size needed to detect a significant difference in effect size, 1 point was added, if not – 0 point). The maximum score in this checklist was 28. The studies were rated as excellent, good, moderate, and poor, based on the percentage of the total score achieved: > 95% (≥ 25), 75–95% (21–24), 55–74% (16–20), and < 55% (≤ 15).

The same reviewers extracted data from the relevant articles, using pre-defined extraction forms, including the aspects of study population, such as mean age and sex, study design, intervention characteristics, follow-up period, and main outcomes. Any disagreements in data extraction were discussed until consensus was reached.

## Results

### Study identification and selection

**Fig. 1:**
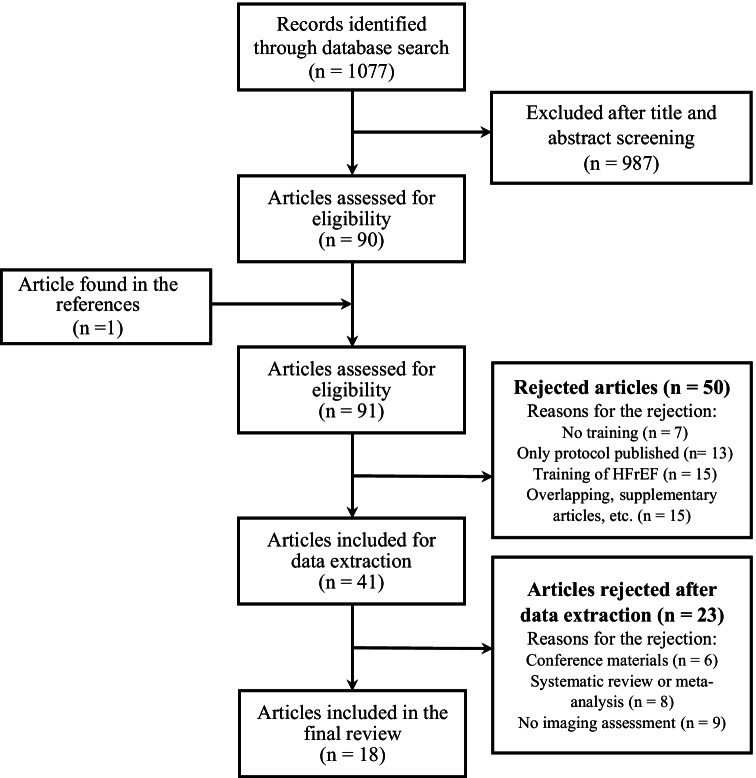
Study flow diagram. This flow diagram shows the selection process of eligible studies

A flow diagram showing the selection of eligible studies is presented in Fig. 1. Initially, our searches in databases identified 1077 relevant publications; after primary review of titles and abstracts, 90 articles were eligible, and one extra paper was later found in the references. After full-text reading, 50 articles were rejected, thus the data were extracted from the rest 41 publications. The reasons for the rejection are described in Fig. 1.

Among these 41 papers, 6 were conference materials and 8 were meta-analyses and (or) systematic reviews—they were removed from the final analyses—leaving 27 articles. Nine from 27 articles did not analyze cardiac imaging parameters at all resulting in a final data set of 18 articles for our systematic review (Fig. 1, Table 2). We contacted the corresponding authors of selected trials asking about any additional findings of echocardiographic, MRI, or invasive measurement parameters; nobody could provide any unpublished data.

**Table 2:** Summary of articles selected and included in the review

**Study (author, year)**	**Duration of the training (weeks)**	**Intervention**	**Trained patients [*N*; mean age, years; male gender,** ***N*** **(%)]**	**Assessed echocardiographic parameters**	**Significant changes of specific echocardiographic parameters**		
**Randomized controlled trials (*N* = 9)**							
Kitzman et al. [68]	16	Outpatient, supervised, endurance training; 60 min 3 times a week	*N* = 26; age = 70 ± 6; male 6 (23.1%) *24 patients in the final analysis*	LV EF, E, A, E/A, DecT, IVRT, LVM, LVV, SV	No significant changes of measured echocardiographic parameters		
Edelmann et al. [30]	12	Outpatient, supervised, endurance training (supplemented with resistance training from the 5th week); 40 min 3 times a week	*N* = 44; age = 64 ± 8; male 20 (45.5%)	LV EF, e' (septal), E/e' (septal), S/D ratio, LAVI, LVMI, LVVI	**↓E/e’** from 12.8 ± 3.2 to 10.5 ± 2.5, change − 2.3 (− 3.0 to − 1.6), *p* < 0.001; between groups − 3.2 (− 4.3 to − 2.1) *p* < 0.001 **↑e’** from 5.4 ± 1.2 to 6.3 ± 1.3, change 0.9 (0.6 to 1.1), *p* < 0.001; between groups 1.2 (0.8 to 1.7) *p* < 0.001 **↓LAVI** from 27.9 ± 7.6 to 24.3 ± 6.5, change − 3.7 (− 4.9 to − 2.4), *p* < 0.001; between groups − 4.0 (− 5.9 to − 2.2) *p* < 0.001		
Alves et al. [31]	26	Outpatient, supervised, endurance training; 35 min 3 times a week	*N* = 20; age = 62.9 ± 10.2; male 22 (71%) *mean age and gender distribution of all of the sample size (trained* + *control)*	LV EF, E/A, DecT, LV dimensions (EDD, ESD)	**↑EF** from 56.4 to 57.7, change 1.3% *p* = 0.01 **↑E/A** from 0.93 to 1.05, *p* < 0.001 **↓DecT** from 236.7 to 222.7, *p* < 0.001		
Smart et al. [27]	16	Outpatient, supervised, endurance training; 30 min 3 times a week	*N* = 12; age = 67 ± 5.8; male 7 (58.3%)	LV EF, E, A, E/A, E/e' (undetermined), DecT, s’, e’ (undetermined), SV, CO, LV-GLS, LV-GSR	**↑CO** from 5.7 ± 2.7 to 7.1 ± 3.1, change 1.4, *p* = 0.04;		
Haykowsky et al. [28]	17	Outpatient, supervised, endurance training; 60 min 3 times a week	*N* = 22; age = 70 ± 6; male 4 (18.2%)	LVV, SV, CO	No significant changes of measured echocardiographic parameters		
Karavidas et al. [69]	6	Outpatient physiotherapy (functional electrical stimulation of the lower limb muscles); 30 min 5 times a week	*N* = 15; age = 69.4 ± 8.6; male 6 (40.0%)	E, A, E/A, E/e' (undetermined), DecT, LAV, a pulmonary − a mitral duration	No significant changes of measured echocardiographic parameters		
Kitzman et al. [70]	16	Outpatient, supervised, endurance training; 60 min 3 times a week	*N* = 32; age = 70 ± 7; male 9 (28.1%) *24 patients in the final analysis*	LV EF, E, A, E/A, DecT, IVRT, LVV, SV	No significant changes of measured echocardiographic parameters		
Palau et al. [71]	12	At home, unattended, inspiratory muscle training; 20 min 2 times a day	*N* = 14; age = 68 [60–76]; male 7 (50.0%)	LV EF, e' (septal), E/e' (septal), LAVI, LVMI	No significant changes of measured echocardiographic parameters		
Palau et al. [32]	12	Home, unattended, inspiratory muscle training; 20 min 2 times a day Outpatient, physiotherapy (functional electrical stimulation of the lower limb muscles); 45 min 2 times a week	IMT (baseline): *N* = 15; age = 75 ± 10; male 7 (46.7%) FES (baseline): *N* = 15; age = 72 ± 9; male 6 (40.0%) IMT + FES (baseline): *N* = 16; age = 73 ± 10; male 8 (50%) *13 patients in each group in the final analysis*	E/e' (undetermined), LAVI	**IMT**: After 12 weeks: **↓E/e’** from 18.4 [14.4–28.0] to 17.2 [12.4–23.1], *p* = 0.015 After 24 weeks^3^: **↓LAVI** from 39 ± 11 to 31 ± 11, *p* = 0.008	**FES**: After 12 weeks: **↓E/e’ from** 20.5 [12–26.4] to 15.7 [11.8–21.8], *p* = 0.001 After 24 weeks^3^: No significant changes of measured echocardiographic parameters	**IMT + FES:** After 12 weeks: No significant changes of measured echocardiographic parameters After 24 weeks^3^: No significant changes of measured echocardiographic parameters
**Randomized parallel group trials (*N* = 5)**							
Yeh et al. [72]	12	Outpatient and home, endurance training compared with Taichi training; supervised 60 min 2 times a week + exercising at home 35 min 3 times a week	AT: *N* = 8; age = 63 ± 11; male 4 (50.0%) Tai Chi: *N* = 8; age = 68 ± 11; male 4 (50.0%)	LV EF, E/A, E/e' (undetermined), LA dimension, LAV	**Tai Chi**^#^: ↓LA dimensions decreased more in the Tai Chi (from 3.8 ± 0.4 to 3.7 ± 0.3) comparing with AT (from 3.7 ± 0.3 to 3.8 ± 0.3), *p* = 0.04		**AT**^#^: ↓E⁄e’ improved more in the AT (from 17 ± 4 to 14 ± 4), comparing with the Tai Chi (from 14 ± 4 to 13 ± 5), *p* = 0.01
Angadi et al. [33]	4	Outpatient, supervised, endurance training; 45 min 3 times a week	HIT: *N* = 9; age = 69.0 ± 6.1; male 8 (88.9%) MI-ACT: *N* = 6; age = 71.5 ± 11.7; male 4 (66.7%)	LV EF, E, A, e' (septal), E/A, E/e' (septal), DecT, IVRT, diastolic dysfunction grade^1^, diastolic dysfunction grade distribution^2^, LAVI	**HIIT:** ↓Diastolic dysfunction grade* from 2.1 ± 0.3 to 1.3 ± 0.7, *p* < 0.01 **↓E from** 0.9 ± 0.3 to 0.8 ± 0.3, *p* = 0.02 **↑DecT** from 194 ± 55 to 225 ± 40, *p* = 0.02		**MI-ACT:** No significant changes of measured echocardiographic parameters
Angadi et al. [29]**	4	Outpatient, supervised, endurance training; 45 min 3 times a week	HIIT: *N* = 9; age = 69 ± 6.1; male 8 (88.9%) MI-ACT: *N* = 6; age = 71.5 ± 11.7; male 4 (66.7%)	LV EF, LVM, LVMI, SV, SVI, RV-GLS, RV-GSR, LV-GLS, LV-GSR	**HIIT:** **↑RV-GLS** from − 18.4 ± 3.2 to − 21.4 ± 1.7, *p* = 0.02		**MI-ACT:** No significant changes of measured echocardiographic parameters
Silveira et al. [36]	12	Outpatient, supervised, endurance training; 38 min (HIIT), 47 min (MCT) 3 times a week	HIIT: *N* = 10; age = 60 ± 10; male 3 (30.0%) MI-ACT: *N* = 9; age = 60 ± 9; male 4 (44.4%)	LV EF, E, A, e' (average), E/A, E/e' (average), DecT, LV dimensions (EDD, ESD), LAVI, LVM, LVVI, LA diameter, SVI	**HIIT:** **↓E/e’** from 13.3 ± 3 to 11.1 ± 2, *p* < 0.001		**MI-ACT:** **↓E/e’** from 14.2 ± 4 to 11.6 ± 3, *p* < 0.001
Mueller et al. [73]	52	Outpatient, supervised (3 months), then continued at home, unattended (for the next 9 months), endurance training; 38 min (HIIT), 47 min (MCT) 3 times a week	HIIT: *N* = 58; age = 70 ± 7; male 17 (29.3%) MI-ACT: *N* = 58; age = 70 ± 8; male 23 (39.7%) *47 patients in the HIIT final and 52 in the MI-ACT final analysis*	E/e' (septal), e' (septal), LAVI	No significant change on measured echocardiographic parameters between HIIT, MI-ACT, and control group^#^		
**Observational trials (*N* = 4)**							
Smart et al. [74]	16	Outpatient, supervised, endurance training (supplemented with resistance training from the 8th week); 60 min 3 times a week	*N* = 18; age = 65 ± 5; male 9 (50.0%)	LV EF, E, A, s’, e’ (average), E/A, E/e' (average), DecT, LVV, LV-GLS, LV-GSR, SV	No significant changes of measured echocardiographic parameters		
Fujimoto et al. [37]	52	Outpatient, supervised, endurance training; 40 min 3 times a week	*N* = 7; age = 74.9 ± 6; male 3 (42.9%)	LV EF, E, A, e' (average), a’, E/A, IVRT, LVV, LVVI	**↑E/A** from 0.75 ± 0.11 to 0.89 ± 0.14, *p* = 0.03		
Nolte et al. [34]*	24	Outpatient, supervised, endurance training (supplemented with resistance training from the 5th week); 30–35 min; 3 times a week	*N* = 24; age = 62 ± 7; male 15 (62.5%)	LV EF, e' (septal), E/e' (septal), S/D ratio, LAVI, LVMI, LVVI	**↓E/e’** from 12.2 ± 3.5 to 10.1 ± 3.0, change − 2.1 (− 3.3 to − 0.9), *p* = 0.002 **↑e’** from 5.9 ± 1.3 to 6.8 ± 1.4, change 0.9 (0.4 to 1.4), *p* = 0.001 **↓LAVI** from 30.0 ± 7.9 to 25.1 ± 8.7, change − 4.9 (− 6.7 to − 3.2), *p* < 0.001		
Fu et al. [35]	12	Outpatient, supervised, endurance training; 30 min 3 times a week	*N* = 30; age = 60.5 ± 2.7; male 20 (66.7%)	LV EF, E/A, E/e’(septal), LV dimensions (EDD, ESD)	**↓E/e ‘** (septal) from 21.0 ± 2.2 to 16.1 ± 1.8, *p* < 0.05		

Data are expressed by number, mean ± standard deviation, median (interquartile range)

*A (m/s)* late mitral inflow velocity, *a’ (m/s)* tissue Doppler mitral annular late diastolic velocity, *AT* aerobic training, *CO (l/min.)* cardiac output, *DecT (ms)* mitral flow E wave deceleration time, *E (m/s)* early mitral inflow velocity, *e’ (m/s)* tissue Doppler mitral annular early diastolic velocity, *E/A* E and A ratio, *EDD (mm)* end diastolic diameter, *E/e’* E and e’ ratio, *FES* functional electrical stimulation, *EF (%)* ejection fraction, *ESD (mm)* end systolic diameter, *HIIT* high-intensity interval training, *IMT* inspiratory muscle training, *IVRT (ms)* isovolumetric relaxation time, *LA* left atrium, *LA dimensions (cm)* the measurement was not clearly defined, *LAV (ml)* left atrium volume, *LAVI (ml/m*^*2*^*)* left atrium volume index, *LV* left ventricle, *LV-GLS (%)* left ventricle global longitudinal strain, *LV-GSR (s*^*−1*^*)* left ventricle global longitudinal strain rate, *LVM (g)* left ventricle mass, *LVMI (g/m*^*2*^*)* left ventricle mass index, *LVV (ml)* left ventricle volume, *LVVI (ml/m*^*2*^*)* left ventricle volume index, *MI-ACT* moderate-intensity aerobic continuous training, *RV-GLS (%)* right ventricle global longitudinal strain, *RV-GSR (s*^*−1*^*)* right ventricle global longitudinal strain rate, *s’(m/s)* tissue Doppler mitral annular systolic velocity, *S/D* pulmonary vein peak systolic velocity and peak diastolic velocity ratio, *SV (ml)* stroke volume, *SVI (ml/m*^*2*^*)* stroke volume index

^#^*p* values comparing the changes between the groups (changes before and after training in separate groups were not published)

^*^In this trial, the same patients that participated in the study of Edelmann et al. [30] were enrolled. Authors presented the same data of trained group changes after 12 weeks of training in both articles, but this article was supplemented by additional data after longer exercise training period (24 weeks); **In this study, the same patients that participated in the study Angadi et al. [33] were assessed, but different echocardiographic parameters were measured—secondary analyses to explore the effects of HIIT on biventricular strain characteristics was carried out

^a^Four grades of diastolic dysfunction were used (0—no diastolic dysfunction, 1—abnormal relaxation pattern, 2—pseudonormal pattern, 3—restrictive filling pattern)

^b^Number of patients in each of the four diastolic dysfunction grades

^c^Follow-up was extended to 24 weeks with the aim of exploring the sustainability of the 12-week training results

### Characteristics of included studies and patients

We extracted the data from nine randomized controlled trials, five randomized parallel group trials (no control, all patients trained, but different training protocols were used), and four observational studies (one group, no control).

All studies included stable patients, diagnosed with HFpEF. The trials were performed in different years (1994–2018) and the definition of HFpEF used in each study varied, but in all trials, LV EF of the participants was ≥ 45%. Echocardiography was applied to measure LV EF in all studies: 7 trials used Simpson biplane, 1—Teichholz method (by M-mode echocardiography), and the remaining 10 studies did not specify the methodology. Detailed information about HFpEF definition used in each study is provided in Supplementary Material (Table S2). All incorporated studies measured at least one cardiac imaging parameter, reflecting LV diastolic function, RV function, or pulmonary hemodynamics.

Our study covered heterogeneous trials with various designs, populations, methodologies, and interventions. The majority of studies were small in sample size—more than 25 patients were trained in only four of the eighteen trials. All training programs were held out-patient, and in sixteen of them, intervention was supervised by healthcare professionals. Eleven programs consisted of endurance training alone, and four, endurance and resistance workouts in combination. Two studies applied resistance training, and in one of them, functional electric stimulation (FES) was added. Only FES was used in a single trial. Various research appraised multifarious echocardiographic parameters and none of them provided random variability in the data for these outcomes. In the selected trials, overall 418 patients (mean age 60.0 to 75.0, 57% female, training duration 4 to 52 weeks) were trained. The components of exercise training and (or) physiotherapy, together with other characteristics and results of these studies, are summarized in Table 2.

The methodological quality of trials, assessed by modified Downs and Black Quality checklist, varied between excellent (*n* = 3), moderate (*n* = 5), good (*n* = 9), and poor (*n* = 1), as summarized in Table 3.

**Table 3:** The quality assessment of included studies by modified Downs and Black Quality checklist

	**Study type**	**Reporting (11)**	**External validity (3)**	**Internal validity**		**Power (1)**	**Total (28)**	Quality^*^
				**Bias (7)**	**Confounding (6)**			
Kitzman et al. [70]	RCT	10	3	6	5	1	25	Excellent
Donelli da Silveira et al. [36]	Randomized parallel group	10	3	6	5	1	25	Excellent
Mueller et al. [73]	Randomized parallel group	10	3	6	5	1	25	Excellent
Kitzman et al. [68]	RCT	10	2	6	4	1	23	Good
Edelmann et al. [30]	RCT	10	1	6	5	1	23	Good
Alves et al. [31]	RCT	10	1	6	5	1	23	Good
Smart et al. [27]	RCT	9	3	5	5	1	23	Good
Palau et al. [71]	RCT	9	2	6	5	1	23	Good
Karavidas et al. [69]	RCT	8	3	5	5	1	22	Good
Palau et al. [32]	RCT	9	1	5	5	1	21	Good
Yeh et al. [72]	Randomized parallel group	9	1	6	5	0	21	Good
Angadi et al. [29]	Randomized parallel group	9	1	5	5	1	21	Good
Haykowsky et al. [28]	RCT	10	1	5	4	0	20	Moderate
Angadi et al. [33]	Randomized parallel group	8	1	4	5	1	19	Moderate
Smart et al. [74]	Observational	10	0	5	1	0	16	Moderate
Nolte et al. [34]	Observational	9	1	5	4	0	19	Moderate
Fu et al. [35]	Observational	9	1	6	3	1	20	Moderate
Fujimoto et al. [37]	Observational	9	1	3	2	0	15	Poor

*RCT* randomized controlled trial

^*^Evaluated by the total score number: ≥ 25 – excellent, 21–24 – good, 16–20 – moderate, ≤ 15 – poor. Studies with no significant changes of assessed echocardiographic parameters after the intervention are marked in gray

### Echocardiographic assessment

All included studies analyzed the changes of echocardiography as secondary endpoints. Various studies appraised multifarious echocardiographic parameters (Table 2). The quantity of studies that assessed specific parameters along with the number of trained patients is shown in Table 4.

**Table 4:** The echocardiographic parameters, assessed in selected studies

**Echocardiographic parameter**	**Number of the studies with assessment of parameter**	**Number of trained patients with assessment of parameter**
**LV EF**	14	292
**E/e’**	12	330
E/e’ (septal)	6	211
E/e’ (average)	2	37
E/e’ (undetermined)	4	82
**e’**	9	261
e’ (septal)	5	205
e’ (average)	3	44
e’(undetermined)	1	12
**LAVI**	7	250
**E/A**	11	210
**E**	8	144
**A**	8	144
**E wave DecT**	8	144
**LVMI**	4	92
**SV**	6	113
**LVVI**	4	87
**LV IVRT**	4	80
**SVI**	3	46
**LV GLS**	3	45
**LV GRS**	3	45
**CO**	2	34
**LV tissue S vel**	2	30
**RV GLS**	1	15
**RV GSR**	1	15

Following parameters were NOT assessed in the included studies: RV diameter, SPAP, TV laterals’, TAPSE, RV FAC, RA area, RA pressure, and IVC diameters

*A (m/s)* late mitral inflow velocity, *a’ (m/s)*, tissue Doppler mitral annular late diastolic velocity, *CO (l/min.)* cardiac output, *DecT (ms)* mitral flow E wave deceleration time, *E (m/s)* early mitral inflow velocity, *e’ (m/s)* tissue doppler mitral annular early diastolic velocity, *E/A* E and A ratio, *E/e’* E and e’ ratio, *EF (%)* ejection fraction, *IVRT (ms)* isovolumetric relaxation time, *LAVI (ml/m2)* left atrium volume index, *LV* left ventricle, *LV-GLS (%)* left ventricle global longitudinal strain, *LV-GSR (s-1)* left ventricle global longitudinal strain rate, *LVMI (g/m*^*2*^*)* left ventricle mass index, *LVVI (ml/m*^*2*^*)* left ventricle volume index, *RA* right atrium, *RV-GLS (%)* right ventricle global longitudinal strain, *RV-GSR (s-1)* right ventricle global longitudinal strain rate, *s’(m/s)* tissue doppler mitral annular systolic velocity, *SPAP (mmHg)* systolic pulmonary artery pressure, *SV (ml)* stroke volume, *SVI (ml/m*^*2*^*)* stroke volume index, *TAPSE (mm)* tricuspid annular plane systolic excursion

As it is shown in Table 2, different studies demonstrated controversial results of the training impact on echocardiographic change. Variations of echocardiographic measurements of trained patients before and after intervention were published in sixteen articles (two trials declared only the changes comparing different training modalities). LV EF and E/e’ were parameters most frequently analyzed—in 14 and 12 studies, respectively.

Five of nine RCTs, four of five randomized parallel group trials, and three of four observational trials reported significant changes of different echocardiographic parameters by training, while the other studies detected no changes in the assessed parameters (Table 2). Significant reduction of mitral E/e’ ratio after the training was reported in 5 of 12 studies, ranging from − 1.2 to − 4.9; significant decrease of LAVI was observed in 3 of 7 trials, ranging from − 3.7 to − 8 ml/m^2^. All but one study showed no significant change of LV EF after the intervention.

Inconsecutive findings were also reported for the change of E/A ratio (9/11 studies showed no change, two statistically significant increase) and E wave DecT (8 studies, one significant increase, one significant decrease).

Furthermore, the impact of exercise training on cardiac output (CO) was reported with inconsistent results, including improvement of CO by 24.5% in one small (*n* = 12), good quality study, after 16 weeks of endurance exercise training, organized 30 min 3 times a week [27]. Another moderate quality trial with older patients (*n* = 22) demonstrated no significant changes of CO after similar duration endurance exercise training, 60 min 3 times a week [28].

The effect of exercising on RV function was assessed in one study [29]. RV global longitudinal strain and RV global longitudinal strain rate were measured before and after 4 weeks of high intensity interval training (HIIT) (*n* = 9) and moderate intensity aerobic continuous training (MI-ACT) (*n* = 6). HIIT group patients demonstrated the increase of RV global longitudinal strain by 3% (from − 18.4 ± 3.2 to − 21.4 ± 1.7), *p* = 0.02. The changes between MI-ACT group patients were insignificant.

As it is shown in Table 4, any other right heart and pulmonary hypertension parameters were not evaluated in the included trials.

Not all studies were well-balanced by the gender of trained participants (Table 4) and neither of them compared echocardiographic changes after the intervention according to sex. However, the majority of trials that revealed significant changes of specific echocardiographic parameters (E/e’, LAVI, DecT, CO, EF, RV-GLS) included predominantly males, or males amounted at least 45% (Table 4) [27, 29–35]. The study of Silveira et al. was the only one with female predominance (63.2%) and significant decrease of E/e’; in this study, a multivariate model was created to adjust E/e’ differences for age, BMI, and sex; the effect of training on E/e’ remained statistically significant after the adjustment [36].

### Invasive hemodynamic assessment

Invasive hemodynamic measurements were performed in only one very small (*n* = 7) poor quality study [37]. Right heart catheterization was performed at baseline and after a year of endurance exercise training. The results revealed that pulmonary artery wedge pressure (PAWP) was unaffected by exercise training in HFpEF patients (16.1 ± 5.6 vs. 15.2 ± 3.6 mmHg, *p* = 0.65). A year of training had no effect on Starling curves (stroke volume index/PAWP) or stroke work-LVEDV relations (as a parameter of LV contractile function), suggesting no change in LV filling and contractile function.

### Cardiac magnetic resonance imaging

None of the eighteen eligible studies assessed the impact of exercise rehabilitation to cardiac magnetic resonance imaging parameters.

## Discussion

To the best of our knowledge, this is the first systematic literature review, which investigated the exercise training and physiotherapy impact not only on LV, but also RV morphology, function, and pulmonary circulation parameters in HFpEF patients. The results of our extended literature review were inconsistent. Eleven of 18 (61.1%) of the considered studies reported a positive impact of exercise training on at least one left and/or right heart function echocardiographic parameter. In seven of all eligible studies (38.9%, five RCTs), no significant changes were observed. Neither of the trials revealed negative effects of exercise training or physiotherapy on heart function (the outcome was positive or indifferent). These findings are in line with previous meta-analyses reporting inconsistent effects of exercise training in HFpEF [12–16].

### LV diastolic dysfunction in HFpEF

Twelve studies assessed mitral E/e′ ratio, and significant decrease of it was observed in five of them [30, 32, 34–36]. Mitral E/e’ ratio is the measure widely accepted as an index of LV filling pressure, but it also has limitations that are relevant in clinical practice, and it is not recommended to use as a single follow-up echocardiographic parameter in HFpEF [38–41].

LAVI is a further echocardiographic parameter, reflecting LV filling pressure, which is crucially important to measure, when assessing LV diastolic function [41]. We found seven articles with published LAVI assessments; three of these studies revealed statistically significant decrease of LAVI after the intervention [30, 32, 34]. Two out of three studies observed significant decrease of LAVI together with substantial decrease of mitral E/e’ ratio, strengthening the tendency of positive training impact to LV diastolic function. When these two parameters were evaluated together, no significant differences of mitral E/e’ ratio or LAVI changes were identified, when comparing the usual care (control) group with any of active treatment groups (IMT; FES; IMT + FES). However, when the analysis of each interventional group was performed separately, significant changes were observed [32]. After 12 weeks of IMT, statistically significant decrease of median mitral E/e’ ratio was observed, while the change of median LAVI was insignificant. After 24 weeks follow-up period, statistically significant decrease of median LAVI was noticed, but the decrease of median mitral E/e’ ratio became insignificant, comparing with the baseline data [32]. These findings could either be explained by the necessity of longer time for the remodeling of LA, as LAVI reflects the cumulative effects of increased LV filling pressures over time [41], or it could be an accidental finding, due to small sample size.

Improvements in mitral E/e’ ratio were more often detected in the trials with larger sample size, which may be a hint that the other studies were underpowered for the effect size of E/e’ changes. Significant decrease in LAVI was more common in studies with a longer follow-up period, which supports the theory of a longer period required to induce reverse atrial remodeling.

Some studies of our systematic review appraised intervention impact on LV diastolic function by evaluating the changes of mitral E wave, A wave, E/A ratio. However, the increase of mitral E wave or E/A ratio can be associated both with improvement and deterioration of diastolic function (a shift from impaired relaxation pattern to normal diastolic function, but also with a shift from normal diastolic function or pseudonormalized pattern to the restrictive filling pattern). For the same reasons and bidirectional interpretation, the changes of mitral E wave DecT and IVRT should not be used for pooled data analysis as well. A solution could be the graduation of LV diastolic function, as it was done by Angadi et al. [33], or use of unidirectional LV diastolic function indices such as mitral e’ and E/e’ ratio.

### RV dysfunction and pulmonary hypertension in HFpEF

Though LV diastolic dysfunction is considered to be the cornerstone of HFpEF, the pathophysiology of the disease is complex. It consists not only of variable contributions of diastolic dysfunction, but also of impaired atrial function, impaired contractile reserve, ventriculo–arterial uncoupling, RV dysfunction, and pulmonary hypertension (PH) [42–44]. Despite variable reports, methods, and criteria, the best available current data indicate that RV dysfunction is present in up to 30–50% of HFpEF. It appears to be present in 18%, 28%, and 21% of HFpEF patients using RV FAC, TAPSE, and RV S’ measurements, respectively [45, 46]. Increased LV filling pressure (> 12 mmHg) in HFpEF promotes symptoms of dyspnea [47], impairs exercise capacity [48], and leads to pulmonary venous congestion and secondary PH, which are associated with worse symptoms and overall prognosis of HFpEF [2]. PH is common in HFpEF—a population-based study reported echocardiographic signs of PH in 83% of HFpEF patients [2]. In a prospective invasive hemodynamic assessment study, 77% of HFpEF patients were diagnosed with PH, and 12% of them had combined post- and pre-capillary PH (CpcPH) [49]. According the recent recommendations to define PH as mean pulmonary artery pressure > 20 mmHg is considered to be abnormal [50]; these numbers probably are even higher.

As LV diastolic dysfunction parameters are not the only ones that are relevant to the symptoms and prognosis of HFpEF patients [2, 44–47, 51, 52], we believe that evaluating effectiveness of exercise training on cardiac mechanisms should not be limited by the estimation of LV function single parameters. Instead, it should be more inclusive, by additionally assessing structural and functional measurements of both atria (LAVI, RA area, RA pressure), LV (LVMI, EDV, ESV, SV), RV, and pulmonary circulation (RV area, RV FAC, TAPSE, RV S’, estimated PAP) and using more precise methods, including 3D and speckle tracking echocardiography. Even applying extended inclusion criteria for the trials, we found very few studies assessing change of indicated LA and LV echocardiographic parameters after the training; almost no studies analyzed specific right heart and pulmonary circulation parameters. This implies the need to evaluate them in the future HFpEF rehabilitation studies.

### Impact of exercise training on HFpEF with PH

There are scientific insights on heterogeneity of HFpEF patients, recommending to look for specific phenotypes [53]. Previously, PH was considered to be limited to the end-stage HFpEF patients, but the study by Borlaug BA et al. revealed abnormalities in PA vasodilation and dynamic RV-PA coupling even in the earliest stages of HFpEF [43]. HFpEF patients with PH (HFpEF-PH) differ in hemodynamics and exercise intolerance, compared with HFpEF patients without PH. Phenotyping HFpEF patients according to the presence of PH in the exercise training studies could be beneficial in gaining a better understanding of the workouts’ role on pulmonary circulation changes and finding the optimal exercise training modality for an individual patient. Significant impact of pulmonary vascular disease on the pathophysiology of exercise intolerance was already proven. During symptom limited peak exercise, CpcPH-HFpEF patients, comparing with non-PH-HFpEF and isolated post capillary PH, demonstrated greater increase in right atrial pressure, enhanced ventricular interdependence, and displayed an inability to enhance cardiac output together with blunted augmentation in RV systolic performance; these changes were coupled with marked limitation in aerobic capacity [20].

### Extra-cardiac mechanisms of exercise intolerance in HFpEF and PH

Though major reasons for reduced physical capacity in many patients with HFpEF seem to be cardiac, non-cardiac factors are also very important. Reduced peripheral oxygen extraction during exercise in these patients was observed [54–56] that can be related to adverse changes in leg muscle mass and volume [57]. The role of extra-cardiac mechanisms of exercise intolerance in PH is probably even more prominent; they include respiratory muscle weakness, dynamic hyperinflation and mechanical constraints [58], poor skeletal muscle and cerebral oxygenation, hyperventilation, and enhanced sympathetic drive [59–61]. Skeletal muscles represent the largest pool of proteins in the organism, and its proper function is essential for locomotion and breathing [62]. Loss of skeletal muscle mass, that is characteristic in advanced HFpEF and PH, directly contributes to exercise intolerance. Exercise training provides benefits at the molecular and physiological level preventing muscle wasting and reduction in force generation [62, 63].

## Limitations

Our systematic review included trials that were conducted in different years (1994–2018). The definition of HFpEF used in each study was not the same. The lowest limit of LV EF in this review was 45%, and according to the very recent ESC guidelines, one of the definition criteria for HFpEF diagnosis is LV EF ≥ 50%, while LV EF 41–49% is considered to be a diagnostic criteria for HFmrEF [4]. Involving a lot of studies with different designs and various statistics, we did not perform a meta-analysis, but previous systematic reviews and meta-analyses of RCT revealed controversial echocardiographic changes after exercise training [12–16]. Potential reasons for these inconsistent results could be related with pooled evaluation of studies with different populations, methodologies, and protocols, when different exercise training modalities and durations of training period and unequal HFpEF diagnostic criteria were used, as well as small sample sizes of the trials. Moreover, the mechanisms of exercise intolerance in HFpEF are complex and the improvement of cardiorespiratory fitness after exercise training might be mediated by cardiac and extra-cardiac mechanisms, being only partially dependent on LV function [28, 64, 65].

## Future research

Our work encourages future HFpEF rehabilitation trials to be supplemented by right heart function and pulmonary circulation evaluation in addition with more precise assessments of LV parameters. Further studies that consider echocardiographic changes after exercising according to sex could be beneficial. Moreover, estimation of specialized rehabilitation influence in HFpEF-PH phenotype would be useful, as until now we have no information about training safety and effectiveness in these patients, while the effectiveness of standardized exercise training in pulmonary arterial hypertension and chronic thromboembolic pulmonary hypertension was already demonstrated [66, 67].

## Conclusions

This systematic literature review that aimed to evaluate and summarize existing data of exercise training and physiotherapy impact on LV, RV morphological, functional, and pulmonary circulation parameters in HFpEF revealed a gap in this area. There are some hypotheses generating findings on potential positive effects on parameters of LV filling pressure (E/e’), left atrial size, cardiac output, and right ventricular function (RV-GLS). However, no reliable evidence about rehabilitation effect to HFpEF cardiac mechanisms is available for now. This encourages a broader and more complex assessment of parameters reflecting cardiac function in the future HFpEF exercise training studies.

## Supplementary Information

Below is the link to the electronic supplementary material.

Supplementary file1 (DOCX 41 KB)
