# Supplementary material for: The effect of exercise training and physiotherapy on left and right heart function in heart failure with preserved ejection fraction: a systematic literature review
Source: Heart Fail Rev. 2022 Jul 13;28(1):193–206. doi: 10.1007/s10741-022-10259-1 (PMC9902326; doi:10.1007/s10741-022-10259-1)
Supplement: Supplementary file 1 — Supplementary file1 (DOCX 41 KB) [file 10741_2022_10259_MOESM1_ESM.docx]

*Supplementary Material for the article “The Effect of Exercise Training and Physiotherapy on Left and Right Heart Function in Heart Failure with Preserved Ejection Fraction: A Systematic Literature Review” submitted to “Heart Failure Reviews” journal by Eglė Palevičiūtė (Clinic of Cardiac and Vascular Diseases, Institute of Clinical Medicine, Faculty of Medicine, Vilnius University, Santariskiu-2, Vilnius 08661, Lithuania. E-mail: egle.paleviciute@santa.lt), Toma Šimbelytė, Christina A. Eichstaedt, Nicola Benjamin, Benjamin Egenlauf, Ekkehard Grünig, Jelena Čelutkienė.*

Table S1. The search strategy for MEDLINE/PubMed database.

| #1 | MeSH descriptor: [Heart Failure] |
| --- | --- |
| #2 | (heart failure) |
| #3 | #1 or #2 |
| #4 | Preserved ejection fraction |
| #5 | Normal ejection fraction |
| #6 | MeSH descriptor: Stroke Volume |
| #7 | Preserved |
| #8 | Normal |
| #9 | #7 or #8 |
| #10 | #6 and #9 |
| #11 | #4 or #5 or #10 |
| #12 | MeSH descriptor: [Exercise] |
| #13 | (exercise) |
| #14 | MeSH descriptor: [Exercise] |
| #15 | (rehabilitation) |
| #16 | MeSH descriptor: [Physical Therapy Modalities] |
| #17 | (physical therapy) |
| #18 | (physiotherapy) |
| #19 | (physical activity) |
| #20 | (physical exercise) |
| #21 | MeSH descriptor: [Physical Fitness] |
| #22 | #12 or #13 or #14 or #15 or #16 or #17 or #18 or #19 or #20 or #21 |
| #23 | #3 and #11 and #22 |

Table S2. HFpEF definitions used in included studies.

| **Study (author, year)** | **Definition of HFpEF used in the study** |
| --- | --- |
| **Randomized controlled trials (N=9)** | |
| Kitzman et al., 2010 [58] | HF symptoms and signs; criteria of Rich et al., that included a history of acute pulmonary edema, or the occurrence of at least 2 of the following with no other identifiable cause: dyspnea on exertion, paroxysmal nocturnal dyspnea, orthopnea, bilateral lower extremity edema or exertional fatigue*, LV ejection fraction ≥50%  Echocardiographic method used for LV EF measurement was not specified. |
| Edelmann et al., 2011 [30] | LV EF > 50%, echocardiographically determined diastolic dysfunction, sinus rhythm, and at least 1 of the following cardiovascular risk factors: overweight, diabetes mellitus, hypertension, hyperlipidemia, smoking.  Echocardiographic method used for LV EF measurement was not specified. |
| Alves et al., 2012 [31] | Definition of HFpEF was not specified.  Echocardiographic method used for LV EF measurement - Simpson biplane. |
| Smart et al., 2012 [27] | Dyspneic patients with LV EF >45% and evidence of either delayed relaxation or pseudonormal filling.  Echocardiographic method used for LV EF measurement was not specified. |
| Haykowsky et al., 2012 [28] | Symptoms and signs of HF defined by The National Health and Nutrition Examination Survey I score ≥3** and the criteria of Rich et al., that included a history of acute pulmonary edema, or the occurrence of at least 2 of the following with no other identifiable cause: dyspnea on exertion, paroxysmal nocturnal dyspnea, orthopnea, bilateral lower extremity edema or exertional fatigue*. LV EF ≥50%, no segmental wall motion abnormalities, and no significant ischemic or valvular heart disease, pulmonary disease, anemia, or other disorder that could explain the patients’ symptoms.  Echocardiographic method used for LV EF measurement was not specified. |
| Karavidas et al., 2013 [59] | Based on the recommendation of the European Society of Cardiology, the inclusion criteria were as follows: (a) symptoms (NYHA class II or III) and signs typical of HF, (b) LV ejection fraction >50% and LV end-diastolic volume index <97 mL/m2, (c) findings of left atrial (LA) dilatation  (LA volume index >40 mL/m2), LV hypertrophy and/or LV diastolic dysfunction (i.e., mitral E/A ratio <1 or >2, mitral E/e’ ratio >15 or 8-15, [A pulmonary – A mitral] duration difference >30 ms,  etc), (d) no alterations in medical therapy during the previous 4 weeks, and (e) no myocardial infarction within 3 months before enrollment. Exclusion criteria included: recent (≤4 weeks)  heart failure decompensation, acute coronary syndrome, chronic inflammatory diseases, and malignancies.  Echocardiographic method used for LV EF measurement was not specified. |
| Kitzman et al., 2013 [60] | Patients had symptoms and signs of HF defined by The National Health and Nutrition Examination Survey I score ≥3** and the criteria of Rich et al., that included a history of acute pulmonary edema, or the occurrence of at least 2 of the following with no other identifiable cause: dyspnea on exertion, paroxysmal nocturnal dyspnea, orthopnea, bilateral lower extremity edema or exertional fatigue*. LV EF ≥50%.  Echocardiographic method used for LV EF measurement - Simpson biplane. |
| Palau et al., 2014 [61] | (a) previous history of symptomatic heart failure (NYHA) functional class II-IV); (b) normal left ventricular ejection fraction (ejection fraction >50% and end-diastolic diameter <60 mm); (c) structural heart disease (left ventricle hypertrophy/left atrial enlargement) and/or diastolic dysfunction estimated by 2D echocardiography;  Echocardiographic method used for LV EF measurement was not specified. |
| Palau et al., 2017 [32] | The diagnosis of HFpEF was made according to the criteria of the 2012 European Society of Cardiology Guidelines: 1. Symptoms typical of HF. 2. Signs typical of HF. 3. Normal or only mildly reduced LVEF and LV not dilated. 4. Relevant structural heart disease (LV hypertrophy/LA enlargement) and/or diastolic dysfunction (reduced e’ <9 cm/s) or an increased E/e’ ratio >15, or a combination of these parameters).  Echocardiographic method used for LV EF measurement was not specified. |
| **Randomized parallel group trials (N=5)** | |
| Yeh et al., 2013 [62] | physician diagnosis of HFpEF; NYHA I, II, or III; LV EF ≥50% (by echocardiography, radionuclide angiography, or contrast angiography) within 2 years of screening.  Echocardiographic method used for LV EF measurement was not specified. |
| Angadi et al., 2015 [33] | Diagnosis of diastolic dysfunction and grade assignment was based on current guidelines for evaluation of left ventricular diastolic function***.  Echocardiographic method used for LV EF measurement was not specified. |
| Angadi et al., 2017 [29] | Diagnosis of diastolic dysfunction and grade assignment was based on current guidelines for evaluation of left ventricular diastolic function***.  Echocardiographic method used for LV EF measurement - Simpson biplane. |
| Silveira et al., 2020 [36] | HF signs and symptoms, LV ejection fraction >50% and evidence of diastolic dysfunction with E/e′ above 15. For those with E/e′ between 8 and 15 and elevation of type B natriuretic peptide (BNP or NT-proBNP) or other diastolic criteria as proposed previously by Heart Failure and Echocardiography Associations of the European Society of Cardiology were considered (Paulus, WJ, Tschöpe, C, Sanderson, JE, et al. How to diagnose diastolic heart failure: A consensus statement on the diagnosis of heart failure with normal left ventricular ejection fraction by the Heart Failure and Echocardiography Associations of the European Society of Cardiology. Eur Heart J 2007; 28: 2539–2550).  Echocardiographic method used for LV EF measurement was not specified. |
| Mueller et al., 2020 [63] | Patients with signs and symptoms of HFpEF (exertional dyspnea [NYHA II-III], LVEF ≥50%, and elevated estimated LV filling pressure (E/e′ medial ≥15) or E/e′ medial of 8 or greater with concurrent, elevated natriuretic peptides (NT-proBNP ≥220 pg/mL or BNP ≥80 pg/mL)  Echocardiographic method used for LV EF measurement was not specified. |
| **Observational trials (N=4)** | |
| Smart et al., 2007 [64] | Dyspneic patients, LV EF >45% and either delayed relaxation or pseudonormal filling.  Echocardiographic method used for LV EF measurement was not specified. |
| Fujimoto et al., 2012 [37] | By Framingham criteria with an ejection fraction >50% documented by transthoracic echocardiography (McKee PA, Castelli WP, McNamara PM, Kannel WB. The natural history of congestive heart failure: the Framingham study. N Engl J Med. 1971; 285: 1441–1446).  Major Criteria  Paroxysmal nocturnal dyspnea or orthopnea  Neck-vein distention  Rales  Cardiomegaly  Acute pulmonary edema  S3 gallop  Increased venous pressure >16 cm of water  Circulation time ≥25 sec  Hepatojugular reflux  Minor Criteria  Ankle edema  Night cough  Dyspnea on exertion  Hepatomegaly  Pleural effusion  Vital capacity ↓ ⅓ from maximum  Tachycardia (rate of ≥ 120/min)  Major or Minor Criterion  Weight loss ≥4.5 kg in 5 days in response to treatment  *For establishing a definite diagnosis of congestive heart failure in this study, 2 major or 1 major & 2 minor criteria had to be present concurrently.  Echocardiographic method used for LV EF measurement - Simpson biplane. |
| Nolte et al., 2014 [34] | NYHA functional class I, II, or III, if they had a preserved left ventricular systolic function (LVEF ≥50%), echocardiographically determined diastolic dysfunction (grade ≥1), and sinus rhythm.  Echocardiographic method used for LV EF measurement was not specified. |
| Fu et al., 2016 [35] | LVEF ≥50% with episodes of acute pulmonary edema after excluding other noncardiogenic etiologies. Hunt SA, Abraham WT, Chin MH, et al: 2009 focused update incorporated into the ACC/AHA 2005 Guidelines for the Diagnosis and Management of Heart Failure in Adults: A report of the American College of Cardiology Foundation/American Heart Association Task Force on Practice Guidelines: Developed in collaboration with the International Society for Heart and Lung Transplantation. Circulation 2009;119:e391Y479  Echocardiographic method used for LV EF measurement: Teichholz (by M-mode echocardiography) |

*Rich MW, Beckham V, Wittenberg C, Leven CL, Freedland KE, Carney RM. A multidisciplinary intervention to prevent the readmission of elderly patients with congestive heart failure. N Engl J Med. 1995 Nov 2;333(18):1190-5. doi: 10.1056/NEJM199511023331806. PMID: 7565975.

**Schocken DD, Arrieta MI, Leaverton PE, Ross EA. Prevalence and mortality rate of congestive heart failure in the United States. J Am Coll Cardiol. 1992 Aug;20(2):301-6. doi: 10.1016/0735-1097(92)90094-4. PMID: 1634664.

***Nagueh SF, Appleton CP, Gillebert TC, Marino PN, Oh JK, Smiseth OA, Waggoner AD, Flachskampf FA, Pellikka PA, Evangelisa A. Recommendations for the evaluation of left ventricular diastolic function by echocardiography. Eur J Echocardiogr. 2009 Mar;10(2):165-93. doi: 10.1093/ejechocard/jep007. PMID: 19270053.
